# Automatic Walking Method of Construction Machinery Based on Binocular Camera Environment Perception

**DOI:** 10.3390/mi13050671

**Published:** 2022-04-25

**Authors:** Zhen Fang, Tianliang Lin, Zhongshen Li, Yu Yao, Chunhui Zhang, Ronghua Ma, Qihuai Chen, Shengjie Fu, Haoling Ren

**Affiliations:** 1 College of Mechanical Engineering and Automation, Huaqiao University, Xiamen 361021, China; 20014080010@stu.hqu.edu.cn (Z.F.); lzscyw@hqu.edu.cn (Z.L.); 19013080044@stu.hqu.edu.cn (Y.Y.); 20014080077@stu.hqu.edu.cn (C.Z.); 20013080034@stu.hqu.edu.cn (R.M.); 11025049@zju.edu.cn (Q.C.); fsj@hqu.edu.cn (S.F.); rhl@hqu.edu.cn (H.R.); 2 Fujian Key Laboratory of Green Intelligent Drive and Transmission for Mobile Machinery, Xiamen 361021, China

**Keywords:** construction machinery, unmanned driving, end-to-end, binocular detection, ranging

## Abstract

In this paper, we propose an end-to-end automatic walking system for construction machinery, which uses binocular cameras to capture images of construction machinery for environmental perception, detects target information in binocular images, estimates the relative distance between the current target and cameras, and predicts the real-time control signal of construction machinery. This system consists of two parts: the binocular recognition ranging model and the control model. Objects within 5 m can be quickly detected by the recognition ranging model, and at the same time, the distance of the object can be accurately ranged to ensure the full perception of the surrounding environment of the construction machinery. The distance information of the object, the feature information of the binocular image, and the control signal of the previous stage are sent to the control model; then, the prediction of the control signal of the construction machinery can be output in the next stage. In this way, the automatic walking experiment of the construction machinery in a specific scenario is completed, which proves that the model can control the machinery to complete the walking task smoothly and safely.

## 1. Introduction

Construction machinery is a basic and strategic industry for the development of the national economy. It has been widely used in industrial construction, and it also plays an irreplaceable role in earthquake-, debris flow-, and other disaster relief work simultaneously [1]. Traditional construction machinery is driven by an internal combustion engine, which generally has problems such as high pollutant emission, a significant amount of noise, and low efficiency [2]. The development of hybrid power and hydraulic energy-saving technologies has greatly improved the efficiency of construction machinery in the past two decades. While solving the above-mentioned problems, the research on electric construction machinery also facilitates the application of various sensors and types of computing equipment in construction machinery [3]. With the gradual popularization of electric construction machinery, intelligence has become the new development trend in construction machinery. Unmanned driving technology provides perception, analysis, reasoning, decision-making, and control functions for construction machinery, which is completely controlled by an unmanned driving system without an operator’s intervention, on all kinds of roads and environments during its operation.

The working environment of construction machinery is harsh, often accompanied by vibration, high temperatures, and dust; and ensures that operators face extremely high risks in their work [4]. Some construction machinery working processes are very repetitive, and tasks such as loading, unloading, and transporting in mines or ports only require the repetition of the same actions in a fixed area. The realization of the near-complete driving automation and the full driving automation of construction machinery can effectively reduce the working risk of construction machinery and reduce labor costs.

Compared with the urban environment, the working scene of construction machinery is more important. In addition, because of the difference between the control systems of passenger vehicles and construction machinery, the control strategies of the two machineries are completely different.

This paper proposes an end-to-end automatic walking system for construction machinery. The system consists of two parts: the binocular recognition ranging model uses binocular cameras to capture images around construction machinery for environmental perception, to detect target information in binocular images, and to estimate the relative distance between the current target and cameras; the control model combines recognition ranging results, image features, and the control signals of the previous stage to predict the instantaneous control signals of construction machinery, to complete driving tasks. 

By improving the network structure of binocular camera detection, we optimized the selection method of candidate targets, designed loss functions, etc., to further improve the accuracy of target detection. Compared with common target detection, our proposed method improves the average accuracy of object detection by more than 10%. In addition, for the special control system of the crawler excavator, we have adopted a control prediction mode that is different from that of the passenger car. We have also changed the input of the predictive control signal network and predicted the control signals of the left and right tracks, respectively, to achieve the control of the vehicle. The system can quickly detect objects within 5 m, which meets the needs of construction machinery working. 

By building an automatic walking system of construction machinery based on binocular camera environment perception, our electric crawler excavator completes the automatic walking task of construction machinery in a specific scenario, which verifies that the system can control the vehicle to complete the walking task smoothly and safely. So, the system can further reduce risks in construction machinery operations and improve labor utilization. The end-to-end system proposed in this paper effectively obtains environmental information, and its structure is relatively simple. Under the complex working conditions of construction machinery, it is easy to realize the automatic walking task of construction machinery, which lays the foundation for construction machinery to perform higher-level intelligent tasks.

## 2. Related Work

### 2.1. Construction Machinery Automation Research

Construction machinery automation research includes two directions: automatic operation and unmanned driving. Additionally, the intelligent construction machinery system framework is proposed by Kim of the Korean Institute of Architecture and Technology and Jeffrey of the University of Wisconsin in the U.S [5]. The framework involves multiple disciplines and multiple systems and requires a high level of performance for system hardware and software. Caterpillar has developed and put into use automatic bulldozers and automatic underground scrapers for mines [6]. These products make full use of the characteristics of high controllability and fixed driving routes in mines, which reduce the difficulty of construction machinery work by curing the operation scenes.

The Australian Robot Center has studied a trajectory planning and control algorithm for automatic working, and the test on the Komatsu mini excavator has proved that the trajectory accuracy can be controlled within 20 cm [7]. The Korean University of Education uses a 3D laser scanner to establish a global model of the construction site and update the working terrain during construction machinery working through building a local model with lidar, but due to the installation height of lidar, the environmental model’s range is limited to 8 m [8]. 

In response to the accuracy problems in the automatic operation of construction machinery, Li Yong from Zhejiang University uses an adaptive echo state network to fit the unknown function of the system on the basis of combining a neural network, adaptive control, and terminal synovial control, so that the control model does not depend on the system model’s parameters [9].

### 2.2. Automatic Walking Control Algorithm

Intelligent construction machinery builds a sensor platform, computing platform, and control platform for sensing, predictive decision-making, and controlling. The sensor platform selects and combines cameras, infrared cameras, and lidar and positioning systems according to the characteristics of the operation scene; the computing platform integrates the environmental information obtained by the sensor platform, makes decisions during its operation [10], and then generates specific control signals during its operation; the control platform usually relies on the existing machinery’s control system.

Decision-making is the core of automatic construction machinery, which determines the operating logic of the entire intelligent system. The traditional rule-based decision-making scheme is easy to tackle with complex function combinations and has good modularity and scalability. However, because the system lacks the depth of scene traversal, it is easy to ignore subtle environmental changes, which easily leads to decision-making errors [11]. With the development of AI technology in recent years, using machine learning to make decisions has become a new trend. The end-to-end decision-making method relies on deep learning to establish a direct mapping from environmental information to control [12], which simplifies the multi-step and multi-module task to a single model, but the method cannot satisfy the decision-making requirements of a complex operating environment. 

**Figure 1:**
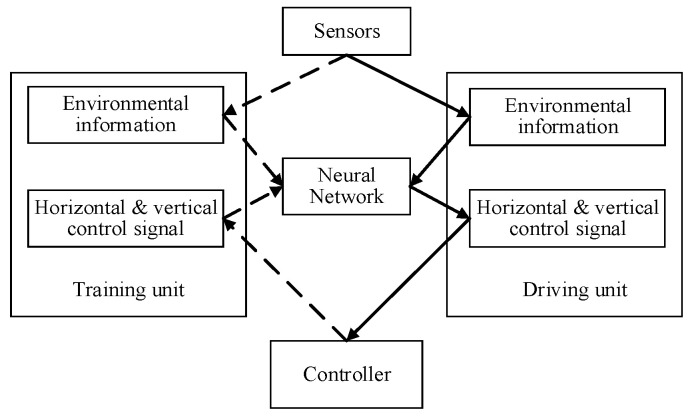
The end-to-end control based on a neural network. The dotted line represents that we can train the network offline to adjust the training weights, which can be used for the real-time detection of the vehicle terminal and output control signals. The solid line part means that our network uses the trained parameters to perform the real-time detection and control the vehicle to walk during the running of the vehicle.

Figure 1 is the schematic diagram of end-to-end control based on a neural network. The end-to-end method uses a deep neural network to fit the complex relationship between input and output, to realize the direct mapping from input data to output results. Through the acquisition of the surrounding environment information by the sensor, and then through the neural network, the control signal of the whole vehicle is predicted and output, and the control signal includes the horizontal control signal and the vertical control signal. The vehicle horizontal signal entails control of the vehicle lateral position error and yaw rate error, and the vehicle vertical signal control refers to control of the vehicle relative distance error and relative speed. Reinforcement learning does not calculate the control information directly, the method turns decision-making into a state-transition problem, and the reward function is used to modify action choices and strategies [13].

### 2.3. YOLOv5

YOLOv5 is an improved version proposed by Ultralytics LLC on the basis of YOLOv4, and it is the most superior one-stage detection network currently [14].

The YOLOv5 network consists of two parts: Backbone and Head. The Backbone part is composed of CONV, Focus, Bottleneck CSP, and SPP modules. CONV is the base layer of the entire network, consisting of a normal convolutional layer, a BatchNormal [15] layer, and a Hardswish activation function [16]; the Focus layer slices and splices the input image, which minimizes the loss of original information; the BottleneckCSP module refers to the CSPNet structure [17], which can reduce the amount of calculation while enriching the gradient combination and avoid duplication in the information integration process by splitting and merging feature maps. The spatial pyramid pooling (SSP) structure [18] uses multi-level spatial bins features to reduce the possibility of information loss during image scaling, which improves the detection accuracy.

Head refers to the FPN structure [19], which transfers and fuses high-level features with low-level features through up sampling and transmits high-level strong semantic features from top to bottom. The PANet [20] connected after FPN carries forward information flow while transmitting low-level strong positioning information from bottom to top. Finally, low-level features and high-level features are concatenated by convolutional layers, which effectively solves multiple-scale issues. 

## 3. Proposed Work

In this section, we present our proposed binocular recognition ranging control system based on the YOLOv5 network structure, which is the end-to-end decision-making method. By improving the network structure, optimizing the candidate target screening method, and designing the loss function, the system can estimate the distance of targets in binocular image and predict the real-time walking control signal of construction machinery.

### 3.1. Binocular Recognition Ranging Control System Based on YOLOv5 Network Structure

With the aim of achieving the walking characteristics of the electric crawler excavator, this paper combines feature extraction and decision-making functions based on the YOLOv5 network structure and establishes direct mapping from the current scene information and the previous cycle control information to the current control signal.

#### 3.1.1. Labels Match with Anchors

YOLOv5 predicts the bounding box by calculating the offset and matching with the anchor in the prediction process [21]. After obtaining the bounding box, the model calculates the loss function, selects candidate boxes through NMS, and finally completes detection. In the training phase, YOLOv5 projects each label box to each feature map’s size and calculates the width and height difference between the label boxes and the corresponding anchor after predicting the offsets.

In the binocular detection ranging model proposed in this paper, each prediction result contains five objects: category, distance, left box, right box, and confidence. There are a total of 11 categories here, which are the label targets determined in advance. The distance here refers to the distance between the target and the camera predicted by the network. The left box and right box mean that we can determine the location of the target in the left and right views, respectively, through the network, and the final confidence describes how reliable we are at producing these predictions. This paper splits predictions during label-matching with anchors, while still using the difference between the width and height to filter the eligible boxes, and expands the left and right targets, respectively, to obtain the most candidate boxes for the loss function calculation.

#### 3.1.2. Non-Maximum Suppression

Non-maximum suppression (NMS) is applied to multiple feature extraction such as data mining and image processing, the essence of which is to suppress non-maximum elements and search for local maximum. As a general algorithm, MNS is mainly used to eliminate redundant candidate boxes and find the best box’s coordinates in detection [22].

**Figure 2:**
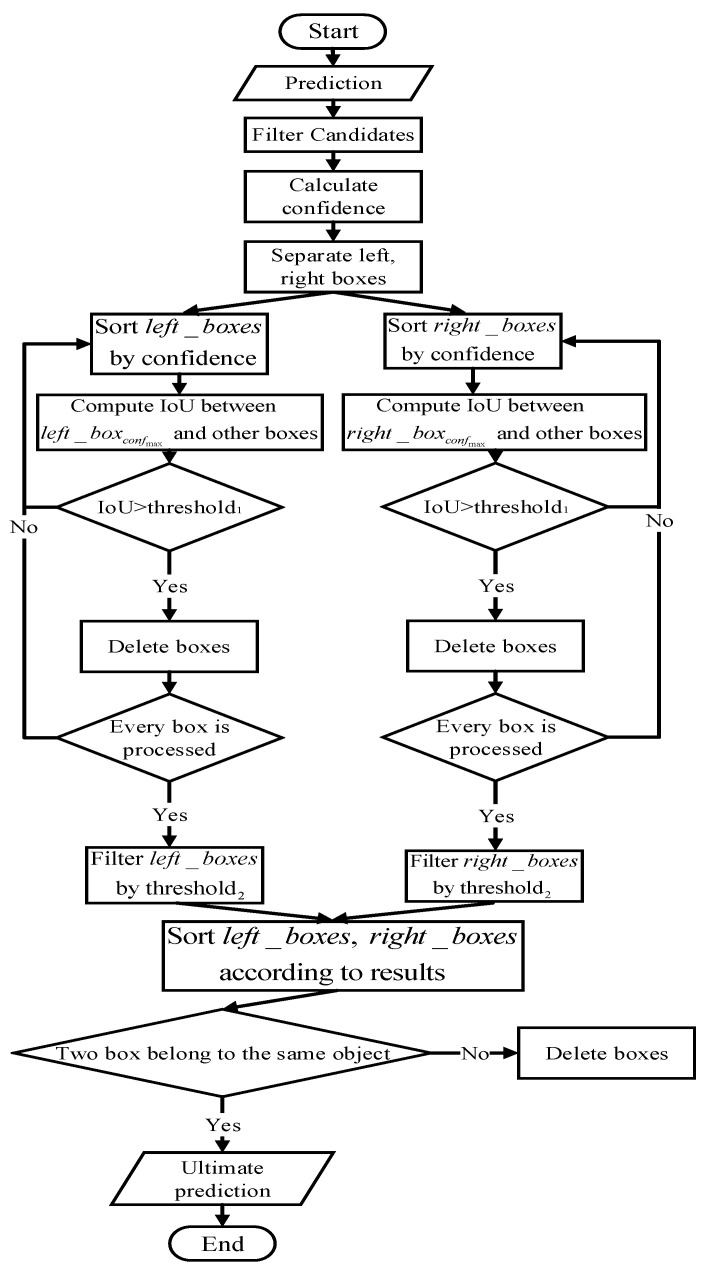
A schematic diagram of dual-target non-maximum suppression. It filters the candidate area from the initial prediction result and then calculates its confidence. Next, the image is divided into two left and right boxes, which, respectively, use the NMS method to filter the candidate area. The network needs to compare the results of the two boxes. Finally, it can predict the candidate box for output.

Every prediction result corresponds to two candidate boxes during binocular object detection. Due to the influence of angle and light, the same object may have large difference between the left and right view in the image. This paper improves NMS for binocular object detection, splits the left and right boxes during filtering, and finally restores the new predictions. The schematic diagram is shown in Figure 2, which filters the candidate area from the initial prediction result and then calculates its confidence. Next, the image is divided into two left and right boxes, which, respectively, use the NMS method to filter the candidate area. The network needs to compare the results of the two boxes. Finally, it can predict the candidate box for output.

### 3.2. Walking Control Signal Prediction Network

**Figure 3:**
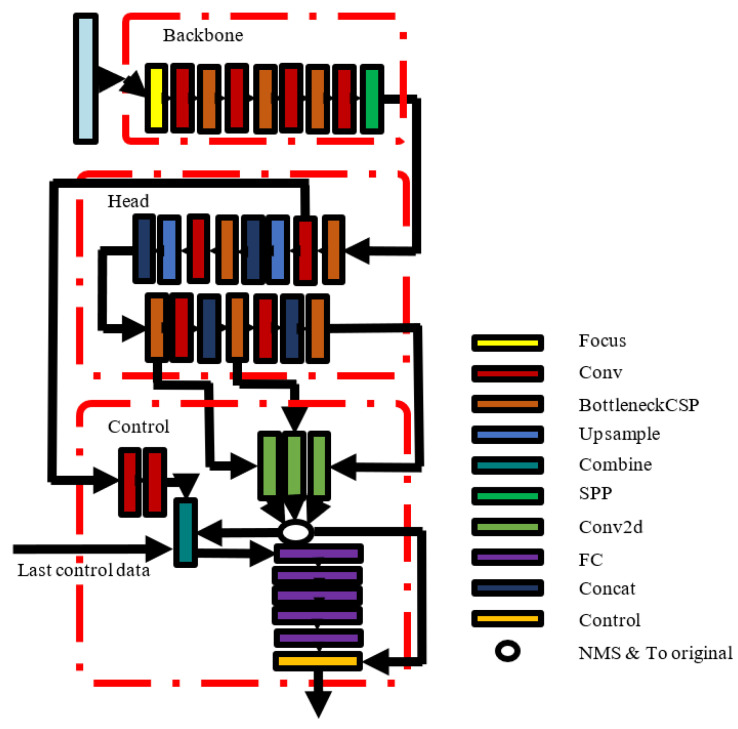
The control neural network structure diagram.

There are many types of construction machinery with different types of vehicle control systems, so it is necessary to design different control signals for automatic construction machinery. We designed a walking control signal prediction network for an electric crawler excavator, which can predict the left and right crawler control signals and thereby complete the walking task of a crawler excavator in fixed scenes. Figure 3 is the diagram of the control signal prediction network structure.

**Figure 4:**
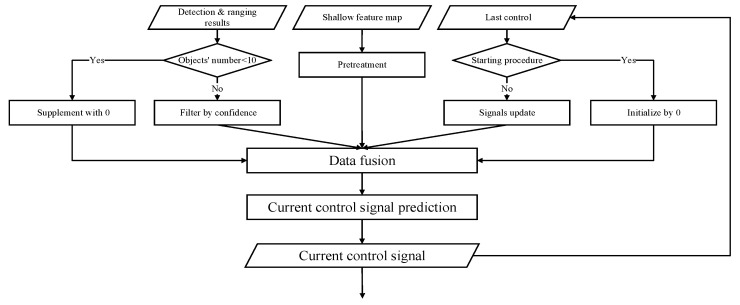
The data update process of the prediction algorithm. Firstly, the model integrates the binocular detection and ranging results that select the top 10 binocular prediction objects sorted by confidence, which will be supplemented with 0 if the screening results are lower than 10. Then, the shallow feature map from the binocular images is entered into the model; meanwhile, we need the control signal of the previous stage as input. Combining these three pieces of information allows us to calculate the control signal at the current moment, and finally it is output.

The walking of construction machinery is a dynamic and continuous process. Due to the different tasks and the initial state of construction machinery, the control signals of construction machinery at the same position in the scene are different. In addition to the current environmental information, the system must introduce information from the previous stage to assist the model in predicting the current control signal. The model integrates the shallow feature map, binocular detection, and ranging results with the control signal of the previous stage input of the control signal prediction network to calculate the control signal at the current moment. The data update process of the prediction algorithm is shown in Figure 4.

The size of the shallow feature map extracted from the backbone network is 512×20×20, which is too large compared with the previous control signal (32×2) and the binocular recognition ranging result (10×27). Using the feature map directly will reduce the influence of the previous stage information and the detection ranging results. The model uses two convolutional layers with batch normalization to reduce the size of the feature map to 128×5×5 before fusing the three kinds of data. The previous stage control signal is the control signals set, composed of the 32 time nodes before the current moment and initialized with 0, which will be updated node by node during the dynamic training and prediction process to ensure the timeliness of the data. The preinstalled detection ranging data are the top 10 binocular prediction objects sorted by confidence, which will be supplemented with 0 if the screening results are lower than 10.

## 4. Binocular Detection Ranging Experiments

### 4.1. Dataset

The dataset is the ceiling of the deep learning algorithm; with the development of unmanned driving technology, a large number of driving environment data sets have emerged. However, compared with conventional roads, there are few data sets for construction machinery scenarios currently. Although the scenarios of construction machinery are changeable and different task scenarios vary greatly, the objects in each scenario, such as the different types of construction machinery, pedestrians, shrubs, and trees usually possess high consistency. It is necessary to build data sets of construction machinery working scenarios.

**Figure 5:**
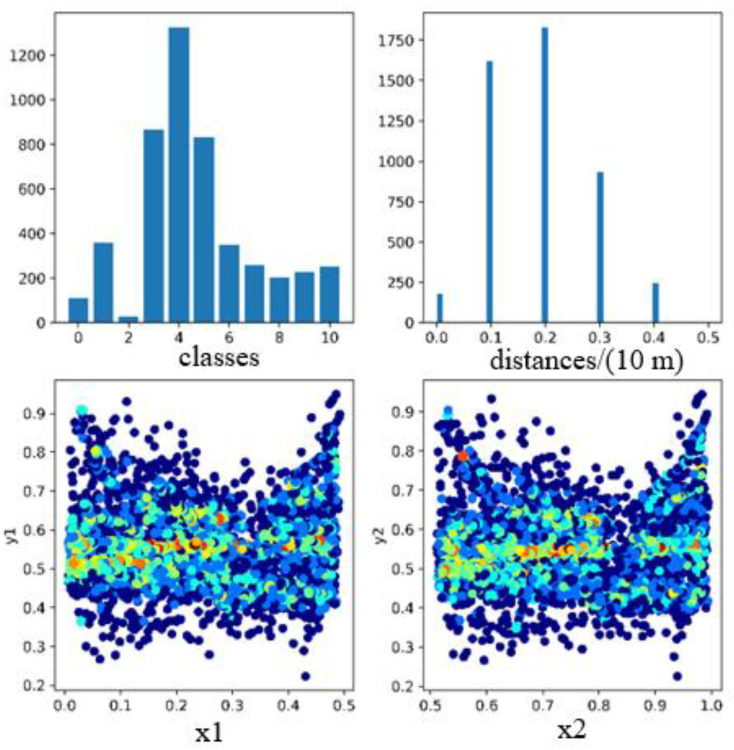
The label data statistics graph. The upper left part shows the statistics of the number of each category. The upper right part shows the statistics of the target distance distribution in the dataset. The lower left and the lower right are the scatter plots of the distribution of the left and right frame targets in the binocular image, respectively.

This paper collects images, the distance information of the objects, and construction machinery walking signals in a construction machinery park at the normal traveling speed (below 5 km/h) of a crawler excavator to construct the dataset. The dataset contains 1007 pictures, including the 11 categories of people, bicycles, motorcycles, trees, shurbs, containers, doors, engineering, steps, the engineering_trail, and the first_landmark. Figure 5 is the statistical graph of the label data. Motorcycle (category2) appears 28 times, which is not obvious in the category statistical graph in the upper left figure. The distance between the objects in the upper right figure is roughly calculated according to the rounding, and the distance distribution of the objects is between 0 and 55 m. The left and right lower pictures count the left and right boxes of the object, respectively, and the left and right boxes are evenly distributed in the left and right views. The dataset is divided into training set and test set according to a ratio of 3:1.

### 4.2. Data Augmentation

A large-scale data set is a guarantee of the accurate prediction of the neural network, but it is impossible to collect a large amount of data in some scenarios due to objective factors; building a data set requires a lot of time and effort, which will affect the performance of model if dirty data is generated in the annotate dataset. Data augmentation is the method of obtaining a large amount of reasonable structure and diverse data through some operations on the original data [23]. It is critical for good performance. 

The original images used in this paper are binocular; in addition to detection, the model also should predict distances and control signals, which are not applicable to augment data through common operations such as inversion, rotation, cropping, and affine transformations. This paper performs random zooming and translations on the image in each round of training and expands the diversity of the data set as much as possible on the basis of ensuring the spatial information of the image. Construction machinery automation research includes two aspects: automatic operation and unmanned driving.

### 4.3. Loss Function and Evaluation Indicator

#### 4.3.1. Loss Function

The loss function is responsible for calculating the difference between the predicted value and ground truth during training. The model uses the gradient back propagation mechanism to adjust the network parameters, reduce the size of the loss function, and optimize the model [24]. The cross-entropy loss function is represented by L_CE (t,p). Its definition is shown as Equation (1), where *x* refers to the total we calculated, which is the total number of targets we need to classify; t(x) is the xth label, which is usually represented by 0 or 1; and p(x) is the xth prediction, which is the probability.

(1) L_CE (t,p)=−∑_x〖(t(x)logp(x)+(1−t(x))log(1−p(x)))〗

Balanced cross-entropy loss function used in this paper introduce α into cross-entropy function to solve the model optimization deviation caused by category imbalance [25]. Here, α is a coefficient that can reduce the inability of the loss function to fall due to the difference in the number of label categories; the balanced cross-entropy loss function’s definition is shown as Equation (2), which is based on the cross-entropy function. It is more suitable for multi-object classification than cross-entropy loss function.

(2) L_BCE (t,p)=−∑_x〖(α∗t(x)logp(x)+(1−t(x))log(1−p(x)))〗

Confidence is an indicator to measure the credibility of prediction. Balanced cross-entropy loss with binary classification form can be used to handle confidence.

The intersection over Union (*IoU*) is the ratio of the intersection and union of the candidate box and the ground truth; its optimization variants are widely used to evaluate detect results and calculate the loss function of candidate boxes. The calculation method is shown as Equation (3), where *A* represents the ground truth and *B* represents the candidate box; the numerator is the intersection of *A* and *B*; and the denominator is the union of *A* and *B*.

(3) IoU=(A∩B)/((A∪B))

The *DIoU* (Distance *IoU*) loss [26] takes into account the influence of distance while having faster convergence speed and higher regression accuracy. Complete-IoU (*CIoU*) introduces an impact factor α on the basis of DIoU, which takes into account the length-to-width ratio of the candidate box to the ground truth and makes the prediction regression better according to overlap area, center point distance, and aspect ratio. The calculation method of *CIoU* is shown in Equations (4)–(6), where b, w, h, b^gt, w^gt, and h^gt represent the center point, width, and height of the candidate box and ground truth. ρ(·) represents Euler’s distance, and c represents the diagonal length of the smallest closed rectangle of the two boxes.

(4) ϑ=4/π^2 〖(arctan w^gt/h^gt−arctan w/h)〗^2

(5) α=ϑ/((1−IoU)+ϑ)

(6) L_CIoU=1−IoU+(ρ^2 (b,b^gt))/((c^2 ) )+γϑ

Unlike the category, confidence, and candidate box, the prediction of the distance information and the control signal is a regression process, using the mean square error (*MSE*) to calculate the error between the predicted value and the true value, and training constantly can improve the predictive power of the model. The calculation method of *MSE* is shown as Equation (7), where t(x) is the xth label and p(x) is the xth prediction, and m is the number of the prediction and label.

(7) L_MSE (t,p)=1/2m ∑_x〖(t(x)−p(x))〗^2 

The loss function of this paper, which is represented by L_2, consists of classification loss, localization loss, confidence loss, ranging loss, and control signals loss; they are represented as 〖L_CE〗_cls, 〖L_CIoU〗_lbox, 〖L_CIoU〗_rbox, 〖L_CE〗_conf, 〖L_MSE〗_ranging, and 〖L_MSE〗_control. The calculation method of loss is shown as Equation (8), where β, γ, θ, δ, and ε are constants balancing the relative importance. We use β=0.5, γ=0.05, θ=1, δ=10, and ε=0 in our binocular detection ranging training process.

(8) L_2=β ∗〖L_CE〗_cls+γ ∗〖〖(L〗_CIoU〗_lbox+〖L_CIoU〗_rbox)+θ ∗〖L_CE〗_conf+δ∗〖L_MSE〗_ranging+ε ∗〖L_MSE〗_control

The weight training process of the binocular detection ranging network is divided into two parts: pre-training and whole-training. Pre-training retains the backbone weight of the YOLOv5m weight and trains the head part of the network for 2000 rounds, and whole-training retains the pre-training weight file to train the overall network for 5000 rounds. The training process uses the cosine annealing method to adjust the learning rate, and the initial learning rate is 0.01.

#### 4.3.2. Evaluation Indicator

The precision and recall are a pair of contradictory measures, which are usually used to evaluate the performance of the detection algorithms. The calculation formulas for precision and recall are shown in Equations (9) and (10), where *P* represents precision, *TP* represents the number of positive samples predicted to be positive, *FP* represents the number of negative samples predicted to be positive, *R* represents the recall rate, and *FN* represents the number of positive samples predicted to be negative:

(9) P=TP/(TP+FP)∗100%

(10) R=TP/(TP+FN)∗100%

### 4.4. Results

#### 4.4.1. Loss Function Analysis

**Figure 6:**
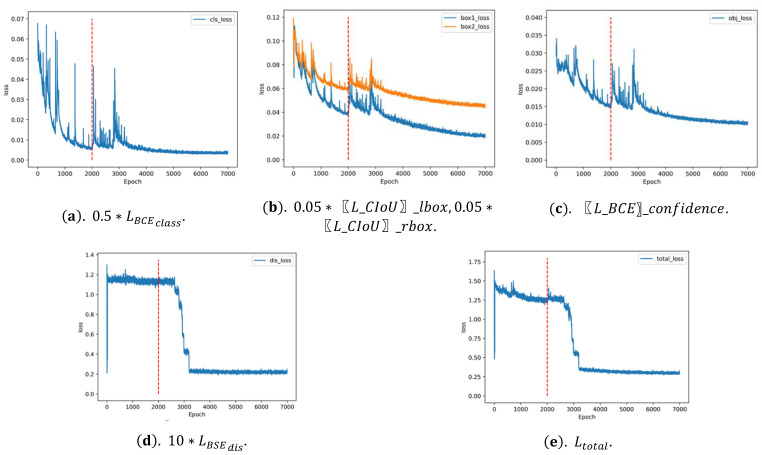
The loss function curve. (**a**) The classification loss curve when *β* is equal to 0.5; (**b**) the localization loss curve, including the left box and the right box when *γ* is equal to 0.05; (**c**) the confidence loss curve when *θ* is equal to 1; (**d**) the ranging loss curve when *δ* is equal to 10; and (**e**) the total loss curve is the sum of the above losses.

This paper retains the backbone part weight of YOLOv5m for migration learning [27] in the pre-training stage and retrains the detection head to obtain weight that is more suitable for binocular detection ranging. After pre-training, the model trains the overall network. Figure 6 is the curves of the loss functions of pre-training and overall training. Due to the change of network structure, the loss function of the entire network training increased sharply in the initial stage and steadily decreased after about 1700 rounds of training.

Classification and confidence prediction are the same as traditional detection tasks, and the loss functions are easier to drop. Data augmentation converts the original input image size of 1280×480 into 640×640 during training and the image becomes 20×20, 40 × 40, and 80×80 after down sampling by 32, 16, and 8, then the model predicts the offsets of these three scales. The image is filled into 672×256 in the test and becomes 21×8, 42×16, and 84×32 after down-sampling. During matching, the predicted offset with the grid converts the left and right candidate boxes to the coordinates of the original image; the second half of the predicted value of the right box offsets obtained from the 32 down-sampled feature map is offset by one grid, which leads to the right box’s loss function always being higher than the left box. The ranging loss function decreases significantly around 3000 rounds and then becomes stable. The overall loss function is greatly affected by the ranging loss function.

#### 4.4.2. Precision-Recall Curve and Detection Ranging Precision Analysis

**Figure 7:**
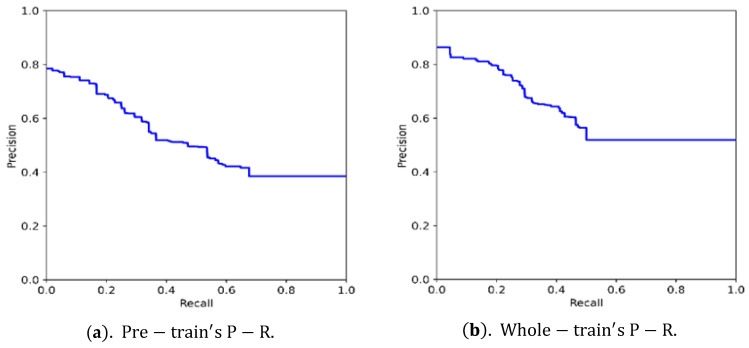
The precision-recall curves. (**a**) The precision-recall curves of the pre-training; (**b**) the precision-recall curves of the overall training.

Select different confidence thresholds from small to large to divide the samples, and calculate the precision P and the recall rate R, respectively, according to Formulas (9) and (10), to obtain a set of points; take P as the ordinate and R as the abscissa, and connect this group point to obtain a P-R curve. Regarding the area enclosed by the P-R curve and the coordinate axis, this can reflect the quality of the model detection. The area is larger, and the model is better. Figure 7 is the precision-recall (P-R) curves of the test set obtained after network pre-training and overall training. The pre-training uses the experience weight effectively through migration training, which accelerates the model fitting speed. The overall network training improves the ability of the model prediction, and the accuracy is significantly improved under the same recall rate.

**Figure 8:**
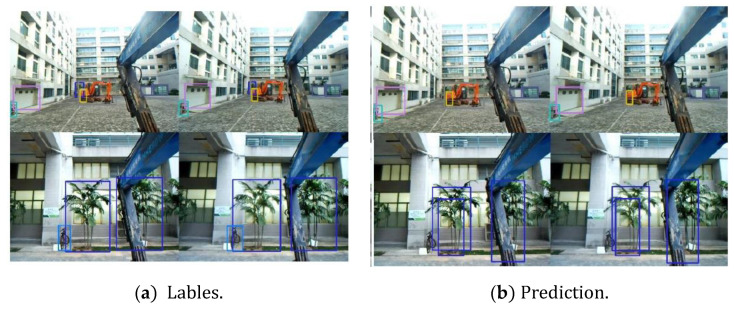
The final detection diagram.

Figure 8 is the final detection diagram of the system. The different colors in the box represent different categories. This paper splits the binocular recognition ranging data set into the left and right views, respectively, for comparison experiments with the YOLOv5 algorithm, and the training process of the model is the same as the binocular detection network. Table 1 is the detection and ranging’s precision of different categories under each model weight. The precision of the model in this paper is significantly improved compared to YOLOv5 because of the dual-target non-maximum suppression with an average precision of 69.62%. The average ranging error of the model is 4.55 m, which can meet the walking demand of construction machinery.

## 5. Control Signals Prediction and Vehicle Test

To verify the automatic working model, this paper modifies an electric crawler excavator and conducts a test with it.

### 5.1. Dataset Automatic Walking Platform of Electric Crawler Excavator

**Figure 9:**
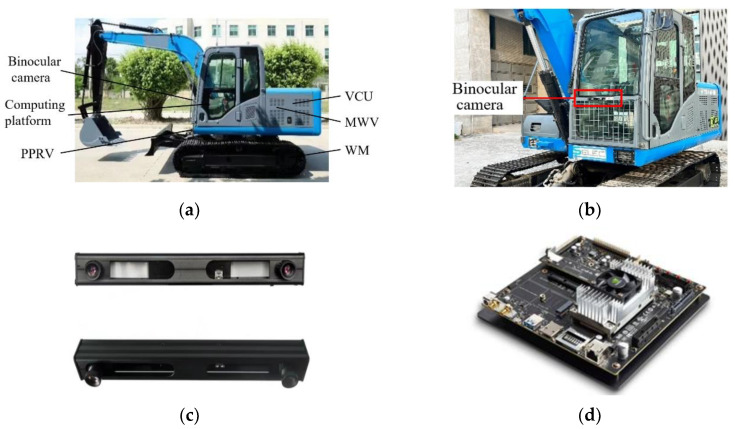
The automatic walking platform of the electric crawler excavator. (**a**) is the arrangement position of various equipment on the crawler excavator; (**b**) is the location where the binocular camera is installed on the crawler excavator; (**c**) is the binocular camera used in the experiment (the camera model is Kingcent); and (**d**) is the computing platform used in the experiment (the model of computing platform is NVIDIA Jetson TX2.5.2. Control Signals Loss).

As shown in Figure 9, the automatic walking platform selects NVIDIA Jetson TX2 as the computing platform. The system obtains a 1280×480 binocular video stream as the input of the algorithm. In order to verify the feasibility of the design principle of the system and simplify the test, the system only installs the binocular camera in the middle of the front of the cab during the test. The construction machinery uses CAN bus communication during its operation. The system converts the prediction to the left and right CAN signals, which are input to the vehicle control unit (VCU) to generate multi-way valve (MWV) pulse signals. The proportional pressure reducing valve (PPRV) outputs the pilot pressure to MWV after receiving the electrical signal, and PPRV controls the left and right walking motors to complete the actions such as steering and moving.

The pre-training of the control model retains the weight of the binocular recognition ranging model and trains the control part of the network for 1000 rounds. Whole-training retains the weight file to train the overall network for 300 rounds. The learning rate setting of this model is the same as the binocular recognition ranging model.

**Figure 10:**
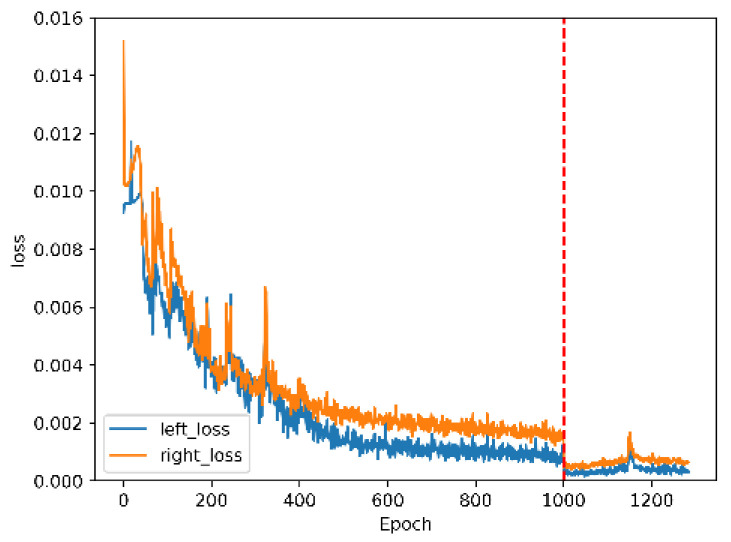
The loss function of the control signals.

Figure 10 is the control signal loss function curve. The control signal loss dropped rapidly during the first 500 rounds of pre-training and then gradually remained stable. Due to the change of the loss parameter, the value of the control loss decreased after entering whole-training, but its true value is still stable with the pre-training loss after stabilization.

### 5.2. Control Signals Prediction and Vehicle Test

**Figure 11:**
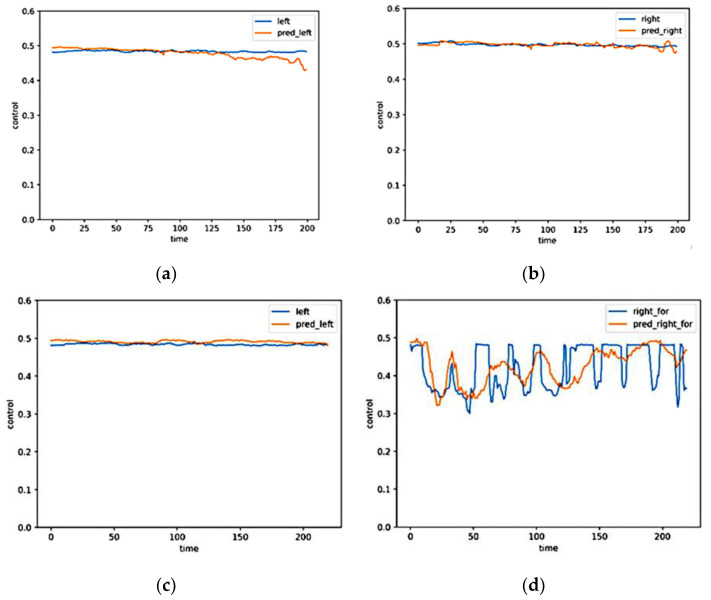
The test sets’ control signal prediction. (**a**) The left signal of test set1; (**b**) the right signal of test set1; (**c**) the left signal of test set2; and (**d**) the right signal of test set2.

Test the model on the straight-line trajectory data set (test set1) and the turning right data set (Test set2). Figure 11 shows the control signal prediction of the system, where the blue curve represents the label value and the yellow curve represents the prediction. Figure 11a,b, respectively, represent the left and right control signals prediction of the Test set1. It can be seen from the figures that the left and right control signals predicted by the system have high accuracy and stability, and there is no forecast surge or sudden decrease. Figure 11c,d, respectively, show the left and right control signals prediction of the Test set2. The data set achieves a right turn through keeping the left control signal stable and adjusting the right control signal constantly. The model achieves good restoration of the relatively stable left control signal, as well as high accuracy and stability; the overall trend of the right control signal prediction is the same as that of the labels, which shows the prediction has better continuity and stability. However, because the right control signal of the labels changes too frequently and lacks continuity, it is difficult to completely restore the label with the predicted control signal.

The system is tested in the operation scene constructed in this paper, and the task is to reach the first_landmark from two different starting points. In test 1, the cab of the electric crawler excavator is facing the first_landmark, and the crawler excavator walks in a straight line to complete the task. The vehicle body is stable during the walking process, and there is no sudden acceleration or deceleration. In Test 2, the first_landmark is located on the right side of the excavator cab, and the vehicle turns slowly during walking. The automatic walking system can identify and autonomously lead to the first_landmark in both tests and complete the driving task successfully. The prediction has better continuity relative to the collected original data; therefore, the vehicle walks more smoothly, which reduces the body vibration, improves the quality of images acquired by the binocular camera, and forms a closed loop to improve the prediction effect.

## 6. Conclusions and Future Work

Intellectualization is one of the development directions of construction machinery. Automatic construction machinery can reduce the risks that are present in the working of machinery and reduce labor costs. The end-to-end system proposed in this paper performs well in terms of obtaining environmental information effectively, which can lead to the realization of the normal walking of construction machinery with good robustness and anti-interference. The working conditions of construction machinery are complex and changeable, and the automatic technology for construction machinery is not perfect. The automatic walking function is the basis for the realization of automatic operation and unmanned driving. On the basis of realizing the automatic walking function, future work can be carried out in the following ways:

1. By obtaining environmental information by using multiple sensors. By adding lidar, MMW radar, the GNSS positioning system, and other equipment to the sensor platform, and by using multi-sensor feature fusion, the spatiotemporal sequence network [28], and other technologies to process image information and point cloud information, the location information can further supplement the spatiotemporal information of the environment and improve the system’s ability to perceive environmental information.
2. By extending data sets. Most of the currently popular environmental data sets are living environment data sets. There are few data sets for the working environment of construction machinery. Building construction machinery working scenarios data sets play a vital role in realizing construction machinery automation.
3. By improving the intelligent system decision-making plan. This paper adopts an end-to-end decision-making method, which has the characteristics of a simplified structure and strong anti-interference. However, the interpretability of this method is low, and it is difficult to modify the model. Introducing rule control and reinforcement learning [29] into the decision-making system can improve the logic of decision-making, make it easier to generalize, and improve its security.

## Figures and Tables

**Table 1:** The detection and ranging precision of the different categories under each model weight.

Loss Function	Binocular-Detection and Ranging			YOLOv5-Lbox (%)	YOLOv5-Rbox (%)
	Pre-Train (%)	Whole-Train (%)	Ranging Error (m)		
Person	66.03	70.10	3.94	56.95	58.59
Bicycle	13.47	56.13	7.32	55.61	43.50
Motorcycle	21.34	29.85	4.43	33.14	30.19
Tree	91.16	93.28	2.13	82.76	86.91
Shrubs	54.74	70.35	3.30	54.45	53.91
Container	83.31	88.88	3.49	67.19	62.06
Door	84.40	86.39	4.01	77.03	77.66
Engineering	70.19	59.90	7.28	63.44	57.10
Step	31.23	53.87	6.16	76.94	84.09
Engineering_trail	38.89	65.43	3.78	50.14	45.76
First_landmark	87.22	92.00	5.29	80.38	70.38
Average	58.36	69.62	4.55	63.46	60.92

## Data Availability

Not applicable.
